# Characterization and Comparative Analysis of Complete Chloroplast Genomes of Three Species From the Genus *Astragalus* (Leguminosae)

**DOI:** 10.3389/fgene.2021.705482

**Published:** 2021-08-05

**Authors:** Chunyu Tian, Xiansong Li, Zinian Wu, Zhiyong Li, Xiangyang Hou, Frank Yonghong Li

**Affiliations:** ^1^Institute of Grassland Research, Chinese Academy of Agricultural Sciences, Hohhot, China; ^2^School of Ecology and Environment, Inner Mongolia University, Hohhot, China; ^3^Key Laboratory of Grassland Resources and Utilization of Ministry of Agriculture, Hohhot, China

**Keywords:** *Astragalus*, complete chloroplast genome, IR lacking, genetic diversity, phylogenetic analysis

## Abstract

Astragalus is the largest genus in Leguminosae. Several molecular studies have investigated the potential adulterants of the species within this genus; nonetheless, the evolutionary relationships among these species remain unclear. Herein, we sequenced and annotated the complete chloroplast genomes of three *Astragalus* species—*Astragalus adsurgens*, *Astragalus mongholicus* var. *dahuricus*, and *Astragalus melilotoides* using next-generation sequencing technology and plastid genome annotator (PGA) tool. All species belonged to the inverted repeat lacking clade (IRLC) and had similar sequences concerning gene contents and characteristics. Abundant simple sequence repeat (SSR) loci were detected, with single-nucleotide repeats accounting for the highest proportion of SSRs, most of which were A/T homopolymers. Using *Astragalus membranaceus* var. *membranaceus* as reference, the divergence was evident in most non-coding regions of the complete chloroplast genomes of these species. Seven genes (*atpB*, *psbD*, *rpoB*, *rpoC1*, *trnV*, *rrn16*, and *rrn23*) showed high nucleotide variability (Pi), and could be used as DNA barcodes for *Astragalus* sp. *cemA* and *rpl33* were found undergoing positive selection by the section patterns in the coded protein. Phylogenetic analysis showed that *Astragalus* is a monophyletic group closely related to the genus *Oxytropis* within the tribe Galegeae. The newly sequenced chloroplast genomes provide insight into the unresolved evolutionary relationships within *Astragalus* spp. and are expected to contribute to species identification.

## Introduction

*Astragalus* is the largest genus in Leguminosae (Li et al., 2014; Su et al., 2021) and is widely distributed in the Northern Hemisphere (Podlech, 1986; Osaloo et al., 2003), South America (Cook et al., 2017a), and Africa (Alami et al., 2019). This genus includes 11 subgenera and some 2000–3000 species (Li et al., 2014), which have been used in various fields. Most *Astragalus* spp. can be used as fresh herbs, forage, or silage (Li et al., 2014), and some have important medicinal values, such as *Astragalus membranaceus* var. *mongholicus* (Lei et al., 2016), whereas some can be toxic and even deadly to humans and livestock, such as *Astragalus miser* var. *oblongitotices* and *Astragalus hamiensis* (Martinez et al., 2019). *Astragalus* belongs to the tribe Galegeae in Papilionoideae; however, it has been a controversial genus concerning its inception, including at the subgenus and species levels. *Astragalus* spp. usually show small, patchy distribution, a pattern that may promote genetic isolation and character differentiation (Massatti et al., 2018). Extensive classical taxonomic studies have explored *Astragalus* spp., based on plant morphology and geography, with many focusing on the discrimination of adulterants (Cui et al., 2012; Zheng et al., 2014; Hou et al., 2016); nevertheless, the systematic evolutionary relationships among *Astragalus* spp. remain unclear.

The chloroplast (cp) is a significant semiautonomous organelle that can absorb carbon dioxide and release oxygen while converting light energy into chemical energy in green plants (Yin et al., 2017), phototrophic bacteria (Trüper, 1987; Mauriello, 2019), and algae (Menke et al., 1965). Chloroplasts can also be used to elucidate the genetic relationships among species and explore plant phylogeny and nuclear evolution (Daniell et al., 2016; Xiong et al., 2020; Zhao and Zhu, 2020), because of its feature of replication initiation, genome stabilization, and maternally-inherited gene conservation (Daniell et al., 2016).

Most complete cp genomes show a typical quadripartite structure with two inverted repeats (IRs) separated by two single-copy regions: a large single-copy region (LSC) and a small single-copy region (SSC). The cp genome usually encodes 120–130 genes with a size of 107–218 kb (Shinozaki et al., 1986; Osaloo et al., 2003; Chumley et al., 2006; Lin et al., 2010; Zha et al., 2020). Although the structure and gene content are relatively stable, divergence has been observed; for example, one copy of the IR was lost in some species, especially in Papilionoideae of Leguminosae, which formed a new clade, named IR lacking clade (IRLC) (Martin et al., 2014; Xiong et al., 2020). Other changes include loss of genes (Palmer and Thompson, 1981; Millen et al., 2001) and inversions (Bruneau and Palmer, 1990).

Since the complete cp genome of tobacco (*Nicotiana tabacum*) was first sequenced and annotated (Shinozaki et al., 1986), an increasing number of cp genomes have been reported. To date, about 26,573 vascular plant cp genomes have been deposited in the National Center for Biotechnology Information (NCBI), including 155 legumes. Within *Astragalus*, complete cp genomes for *Astragalus* laxmannii (Liu et al., 2020), *A. membranaceus* (Lei et al., 2016), *A. mongholicus* var. *nakaianus* (Choi et al., 2016), *A. membranaceus* var. *membranaceus* (Wang et al., 2016), *Astragalus* strictus, and *Astragalus* gummifer have been sequenced and annotated; however, the latter two can only be found in NCBI. It should be noted that, except *A. laxmannii*, all the other five species only have one copy of the IR region, but they all belong to the IRLC. Moreover, they have a different phylogenetic relationship with other species concerning morphological taxonomy (Choi et al., 2016; Lei et al., 2016; Wang et al., 2016; Liu et al., 2020), which further proves the controversy regarding *Astragalus* taxonomy.

*Astragalus adsurgens*, *A. mongholicus* var. *dahuricus*, and *A. melilotoides* belong to three different subgenera (subg. Cercidothrix, subg. Trimeniaeus, and subg. Phaca, respectively) of *Astragalus*; however, many of the subgenera of *Astragalus* are not monophyletic and their phylogenetic relationships within the genus are still poorly known (Su et al., 2021). Recent studies have shown that the taxonomic classifications within the genera based on morphology do not correspond to the phylogenetically recovered clades (Tunckol et al., 2020). Moreover, it is unclear why *Astragalus* and its clades have such a high number of species (Bagheri et al., 2017). Therefore, we sequenced and annotated the complete chloroplast genome of *A. adsurgens*, *A. mongholicus* var. *dahuricus*, and *A. melilotoides* to explore the relationships among *Astragalus* species. Then, repetitive sequences, simple sequence repeats (SSRs), nucleotide diversity (Pi), and evolution were investigated. In addition, a phylogenetic tree was constructed using the information from 37 species to examine their evolutionary relationships.

## Materials and Methods

### Plant Materials

Young leaves of *A. adsurgens*, *A. mongholicus* var. *dahuricus*, and *A. melilotoides* were collected at Hohhot, Inner Mongolia, China (40.57°N, 111.93°E) and deposited at the National Germplasm Perennial Herbage Nursery, Institute of Grassland Research, Chinese Academy of Agricultural Sciences.

### DNA Extraction and Sequencing, Genome Assembly, and Annotation

Genomic DNA was extracted from fresh leaves using a Plant DNA Isolation Kit (Tiangen, Beijing, China) and sequenced using the MiSeq PE150 platform (Illumina, San Diego, CA, United States), yielding 150 bp paired-end reads, at Novogene Co. (Tianjing, China). The cp genome was *de novo* assembled using NOVOPlasty (Dierckxsens et al., 2019) with default parameters. Genomes were annotated using the plastid genome annotator (PGA) tool (Qu et al., 2019), coupled with manually edited start and stop codons using Geneious (Kearse et al., 2012). *A. mongholicus* cp genome sequence (NCBI accession number: NC029828) was used as a reference. The annotation results were checked using the Dual Organellar GenoMe Annotator (DOGMA) (Wyman et al., 2004) and CpGAVAS2 (Shi et al., 2019). OGDRAW^1^ (version 1.3.1) (Greiner et al., 2019) was used to draw the gene map of the cp genomes.

### Identification of Repeat Sequences and Simple Sequence Repeats

REPuter software (Kurtz and Schleiermacher, 1999) was used to identify repeat sequences, including forward repeat (F), reverse repeat (R), complementary repeat (C), and palindromic repeat (P) in cp genomes. Detection parameter settings were as follows: minimum repeat size 30 bp and an edit distance of 3. The MIcroSAtellite identification tool (MISA^2^) was used for SSR identification on the cp genome sequences with the following parameter settings: unit size (nucleotide) _min-repeats: 1_8, 2_5, 3_4, 4_3, 5_3, and 6_3. The minimum distance between two SSRs was set to 100 bp.

### Polymorphism Analysis and Genome Structure Comparison

Pi values and sequence polymorphisms of eight *Astragalus* species were analyzed using DNAsp v. 6.10 (Rozas et al., 2017). mVISTA (Frazer et al., 2014) software was used to compare the complete cp genomes of *A. adsurgens*, *A. mongholicus* var. *dahuricus*, and *A. melilotoides* that we sequenced, with four additional published cp genomes of congeneric species (*A. gummifer*, *A. mongholicus*, *A. nakaianus*, and *A. strictus*) with the shuffle-LAGAN mode and *A. membranaceus* var. *membranaceus* annotation (Wang et al., 2016) as reference.

### Gene Selective Pressure Analysis

To detect whether cp genes were under selection pressure, synonymous (dS) and non-synonymous (dN) substitution rates, and the ω value (ω = dN/dS) for shared protein-coding gene in eight *Astragalus* cp genomes were analyzed using Phylogenetic Analysis by Maximum Likelihood 4.0 with the YN algorithm (Yang, 2007).

### Phylogenetic Analysis

The three sequenced cp genomes of *Astragalus*, along with the genomes of 34 species (using *Lotus japonicus and Glycine max* as outgroups) retrieved from NCBI, were used to construct a phylogenetic tree. Multiple alignments were performed using complete cp genomes based on the conserved structure and gene order, and all nucleotide sequences were aligned using the multiple sequence alignment MAFFT software (Katoh and Standley, 2013) with default parameters. Two methods, maximum likelihood (ML) and Bayesian inference (BI), were employed to construct the phylogenetic trees. ML analyses were conducted using RAxML 8.2.11 (Stamatakis, 2014) with the GTR + Gamma nucleotide substitution model; node support was conducted by a bootstrap analysis with 1000 replicates. BI analyses were conducted using MrBayes v. 3.2.6 (Ronquist and Huelsenbeck, 2003).

## Results and Discussion

### Characteristics of *A. adsurgens*, *A. mongholicus* var. *dahuricus*, and *A. melilotoides* Complete Chloroplast Genomes

**FIGURE 1:**
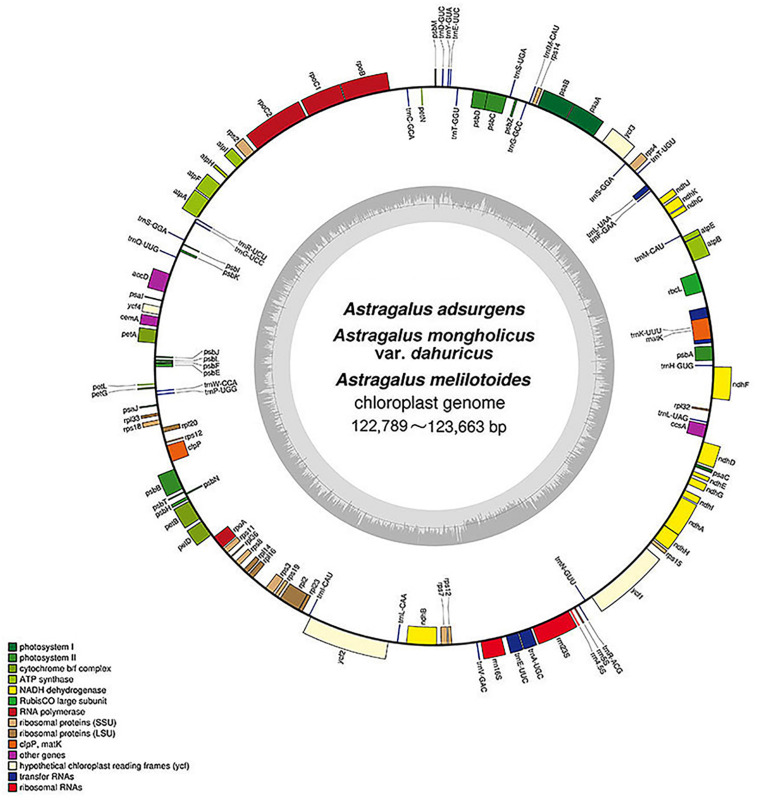
Structure and characteristics of the complete chloroplast genomes of three *Astragalus* species. Genes inside of the circle are transcribed in the clockwise direction and those outside the circle are transcribed in the counter-clockwise direction. Different colors indicate genes belonging to different functional groups. Dark gray in the inner circle indicates the GC content, and light gray indicates AT.

In the present study, we sequenced and annotated the complete cp genomes of three *Astragalus* species—*A. adsurgens*, *A. mongholicus* var. *dahuricus*, and *A. melilotoides*. The general gene structure and locations in the cp genomes are presented in Figure 1. All genomes were found to have lost one copy of the IR region, thereby being affiliated to IRLC in Papilionoideae, and showed the same GC content of 34% (Figure 1). The cp genomes were 122,796, 122,789, and 123,663 bp for *A. adsurgens*, *A. mongholicus* var. *dahuricus*, and *A. melilotoides*, respectively. *A. melilotoides* and *A. adsurgens* consisted of 106 genes including 76 protein-coding genes, four rRNAs, and 26 tRNAs; *A. adsurgens* had one tRNA (*trnE-UUC*) gene copy, whereas *A. melilotoides* and *A. mongholicus* var. *dahuricus* had two. Only *A. mongholicus* var. *dahuricus* had *trnG-UCC* and *trnK-UCC* in its genome. The species lacked *trnfM-CAU* and *trnS-GGA*, found in the cp genomes of *A. adsurgens* and *A. melilotoides*, which were replaced by *trnM-CAU* and *trnS-GCU* in the *A. mongholicus* var. *dahuricus* chloroplast genome. Thus, *A. mongholicus* var. *dahuricus* cp genome consisted of 108 genes. The numbers of tRNAs in the three species differ from those in other *Astragalus* spp. (Choi et al., 2016; Lei et al., 2016; Wang et al., 2016).

Among the genes in the cp genome, 45 were related to photosynthesis, including five subunits of photosystem I, 16 subunits of photosystem II, six subunits of ATP synthase, 11 subunits of NADH-dehydrogenase, and six subunits of cytochrome b/f complex as well as *rbcL* (a subunit of Rubisco). Genes related to self-replication included eight large subunits of ribosome, 11 small subunits of ribosome, and four DNA-dependent RNA polymerases. Genes related to self-replication were also detected, including four ribosomal RNAs, *rrn5S*, *rrn4.5S*, *rrn16S*, and *rrn23S*. In particular, there were five other genes and three genes, *ycf1*, *ycf2*, and *ycf4*, whose functions are unknown (Table 1). The structures and locations of the genes are shown in Figure 1. In comparison with other angiosperm plastid genomes, all three species lost *rps16*, *rpl22*, and *infA*, consistent with the *A. membranaceus* cp genome (Cook et al., 2017b). However, *rps16* and *rpl22* could be found in most angiosperm cp genomes (Shen et al., 2018; Biju et al., 2019; Liu et al., 2019). Their absence in the three species may be explained by genome rearrangement during the evolution process or elimination by natural selection (Daniell et al., 2016). In some species, *infA* has been transferred from the chloroplast to the nuclear genome (Millen et al., 2001); thus, it is reasonable to infer that lack of *infA* in the cp genome of the three species may be explained by a similar process. However, further studies are needed to evaluate this hypothesis. Overall, 12, 11, and 11 genes in the cp genomes of *A. adsurgens*, *A. mongholicus* var. *dahuricus*, and *A. melilotoides*, respectively, contained one intron. In addition, *ycf3* had two introns in the *A. adsurgens* and *A. mongholicus* var. *dahuricus* cp genomes. In *A. melilotoides*, *trnL-UAA* had two introns (Table 1 and Supplementary Table 1).

**TABLE 1:** List of genes encoded by three species of *Astragalus*.

Category of genes	Group of genes	Genes
Genes for photosynthesis (45)	Subunits of photosystem I	*psaA, psaB, psaC, psaI, psaJ*
	Subunits of photosystem II	psbA, psbB, psbC, psbD, psbE, psbF, psbI, psbJ, psbH, psbK, psbL, psbM, psbN, psbT, psbZ, ycf3**
	Subunits of ATP synthase	atpA, atpB, atpE, atpF*, atpH, atpI
	Subunits of NADH-dehydrogenase	ndhA*, ndhB*, ndhC, ndhD, ndhE, ndhF, ndhG, ndhH, ndhI, ndhJ, ndhK
	Subunits of cytochrome b/f complex	petA, petB*, petD*, petG, petL, petN
	Subunit of Rubisco	*rbcL*
Self-replication (55)	Large subunit of ribosome	rpl14, rpl16, rpl2*, rpl20, rpl23, rpl32, rpl33, rpl36
	Small subunit of ribosome	*rps11, rps12, rps14, rps15, rps18, rps19, rps2, rps3, rps4, rps7, rps8*
	DNA-dependent RNA polymerase	rpoA, rpoB, rpoC1*, rpoC2
	Ribosomal RNAs	*rrn5S, rrn4.5S, rrn16S, rrn23S*,
	tRNA genes	*trnH-GUG, trnM-CAU, trnF-GAA, trnL-UAA, trnT-UGU, trnS-GGA, trnfM-CAU, trnG-GCC, trnS-UGA, trnT-GGU, trnE-UUC(× 2), trnY-GUA, trnD-GUC, trnC-GCA, trnR-UCU, trnS-GGA, trnQ-UUG, trnW-CCA, trnP-UGG, trnI-CAU, trnL-CAA, trnV-GAC, trnA-UGC, trnR-ACG, trnN-GUU, trnL-UAG, trnG-UCC, trnK-UCC*
Other genes (5)	Subunit of acetyl-CoA-carboxylase	*accD*
	c-type cytochrome synthesis gene	*ccsA*
	Envelop membrane protein	*cemA*
	Protease	clpP*
	Maturase	*matK*
Genes with unknown function (3)	Conserved open reading frames	*ycf1*, *ycf2*, *ycf4*

* and ** indicate genes containing one/two introns.

### Repeat Sequences and SSRs Analysis

**FIGURE 2:**
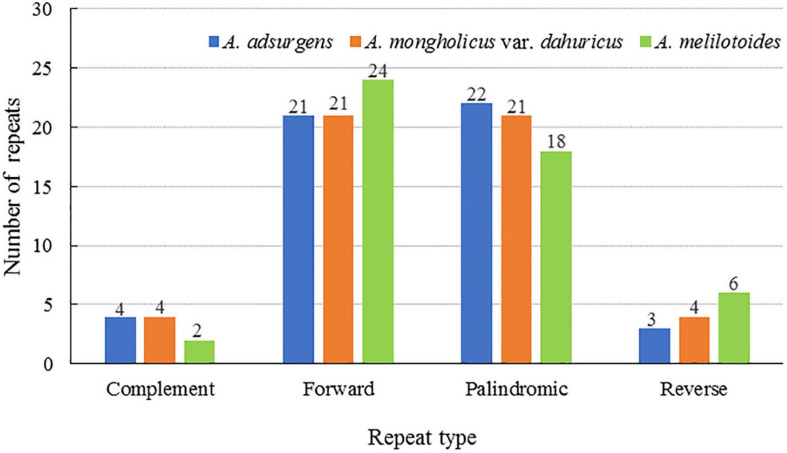
Number and proportion of repetitive sequences in the cp complete genomes of three *Astragalus* species.

Repetitive sequences are the primary source of repeat, deletion, and rearrangement events in the chloroplast genome (Li and Zheng, 2018). Furthermore, nuclear and genome rearrangements contribute to the majority of repetitive sequences. Herein, 50 scattered repetitive sequences with lengths of no more than 30 bp, including forward, reverse, complementary, and palindromic repeats, were detected in the three species of *Astragalus*. The proportions of each type of repetitive sequence differed slightly among species. In the *A. adsurgens* cp genome, palindromic repeats were the most common (44%), followed by forward (42%), complementary (8%), and reverse (2%). Equal numbers of forward and palindromic (42%) as well as of complementary and reverse repeats (8%) were detected in *A. mongholicus* var. *dahuricus* genomes. Forward (48%) was the most common type of repeat in the *A. melilotoides* cp genome, followed by palindromic (36%), reverse (12%), and complimentary (4%) repeats (Figure 2). Those with lengths of 30–40 bp accounted for the majority of repetitive sequences (Supplementary Table 2). Compared with *A. membranaceus* (Lei et al., 2016), all three species in this study lacked tandem repeat sequences, suggesting that the mutation frequencies and rate of evolution are high in *A. membranaceus* (Saltonstall and Lambertini, 2012).

**FIGURE 3:**
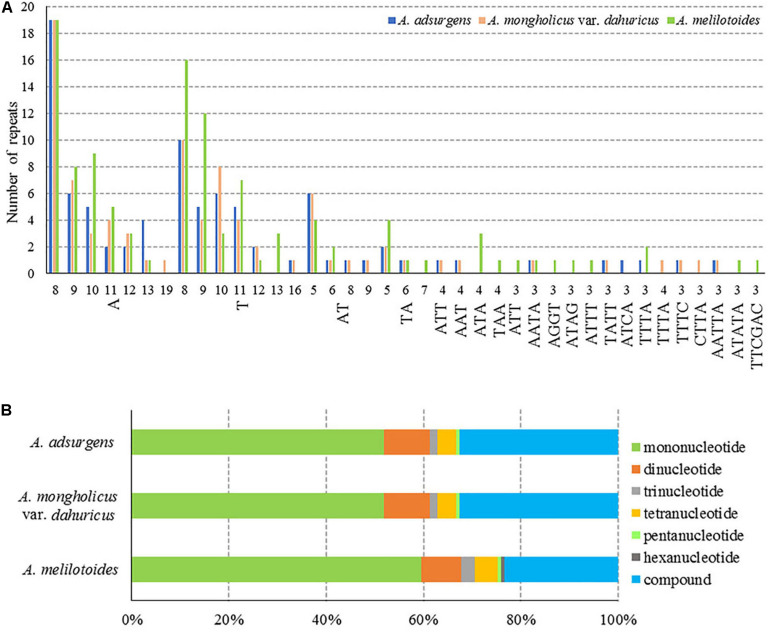
Numbers and proportions of repetitive sequences in the cp complete genomes of three *Astragalus* species. **(A)** Number of SSR motifs of different repeat types. **(B)** Number of repeat sequences.

Molecular markers can be used for genome mapping, identification of genetic relationships, and systematic classification of species (Kapoor et al., 2020). Among different types of DNA molecular markers, SSRs are highly polymorphic, codominant, and widely distributed across genomes and therefore are useful for studies of genetic diversity and relationships among plant populations (Saha et al., 2019; Li et al., 2020). The chloroplast SSRs (cp SSRs) are maternally inherited, thus they are considered to be highly efficient tools in the studies of population structure, genetic variation, species identification, and phylogenetic relationships analyses (Saski et al., 2005). In particular, 146 SSRs (8–298 bp) were detected in the cp genome of *A. melilotoides*, and 129 SSRs (8–335 bp) were detected in the *A. adsurgens* and in the *A. mongholicus* var. *dahuricus* cp genomes. The same number of SSRs can also be found in *Lupinus albus* and *Lupinus luteus* (Zha et al., 2020). In addition, the numbers of mononucleotide, dinucleotide, trinucleotide, tetranucleotide, and pentanucleotide repeats were the same in the *A. adsurgens* and *A. mongholicus* var. *dahuricus* cp genomes, which had no hexanucleotides; however, the types were slightly different (Figure 3A and Supplementary Table 3). Among the three species, mononucleotides were the most frequent repeat type, and most of them were A/T homopolymers, accounting for 59.59% of all SSRs in *A. melilotoides* and 51.94% in *A. adsurgens* and *A. mongholicus* var. *dahuricus* cp genomes. There were 12 dinucleotides in three species, which were AT/TA or TA/AT, accounting for 8.22–9.30% of the SSRs, and no more than four trinucleotides and seven tetranucleotides in the three complete cp genomes. All the species had one pentanucleotide, and only *A. melilotoides* had one hexanucleotide. The cp SSRs identified in the species, mainly poly-A/T and C/G, are rare, even for multiple base repeats. These results are consistent with those for most species sequenced in IRL clade in Papilionoideae (Lei et al., 2016; Liu et al., 2016; Somaratne et al., 2019; Wei et al., 2020). Furthermore, compound SSRs accounted for 23.56–32.56% of the three cp genomes. Although the richness of SSRs was similar within *Astragalus*, the differences in SSR count may be a useful molecular marker for species identification (Figure 3B and Supplementary Table 3). However, using SSRs to elucidate ecological and evolutionary processes has yet to be fully achieved (Ebert and Peakal, 2009). The herein described SSRs in the cp genomes of *Astragalus* may pave the way for exploring evolutionary processes at the population level.

### Comparative Genome Analysis and Sequence Variation

**FIGURE 4:**
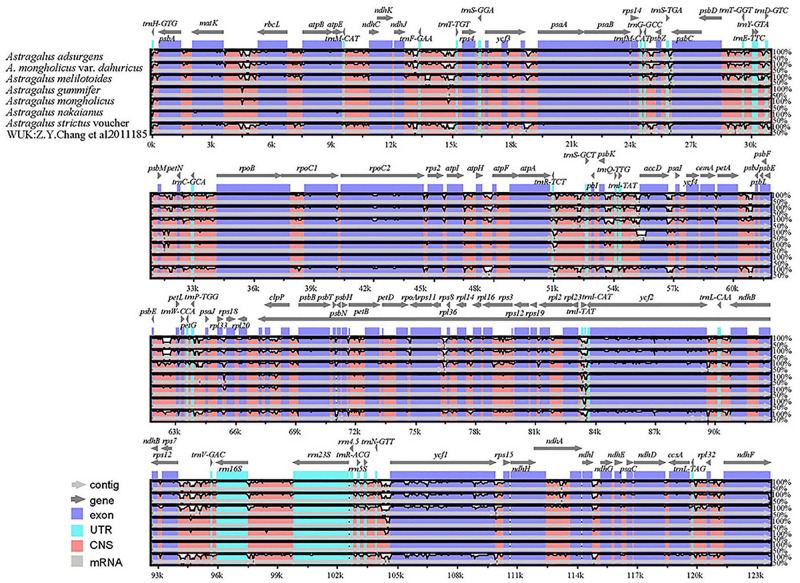
Global alignment of seven cp genomes of *Astragalus* generated using mVISTA. The *y*-axis represents the range of identity (50–100%). The *x*-axis indicates the coordinate in the cp genome. Annotated genes are shown along the top. The alignment was generated using *A. membranaceus* var. *membranaceus* as reference. Genomic regions are color-coded to indicate protein-coding regions, exons, UTRs, and CNS.

**FIGURE 5:**
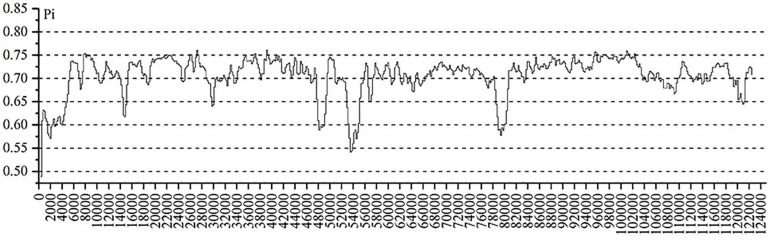
Nucleotide diversity (Pi) among cp genomes of four *Astragalus* species.

The highly variable regions of the cp genome can be used to identify closely related species and provide abundant information for further phylogenetic studies (Cui et al., 2020). Setting *A. membranaceus* var. *membranaceus* as reference, we used mVISTA to compare the cp genomes of seven species of *Astragalus* species, including the newly sequenced genomes and data deposited in the NCBI database, to explore sequence variation (Figure 4). The cp genome length varied among species, being *A. mongholicus* var. *dahuricus* genome (122,789 bp) the shortest and that of *A. nakaianus* (123,633 bp) the longest. In general, there was high sequence similarity among the cp genomes of the seven species, with high conservation of size and gene order. However, sequence variation was higher in conserved non-coding sequences (CNS) regions than in other regions. In addition to start–*trnH–GTG*, *atpE–trnM–CAT*, *trnT–TGT–rps4*, *rps14–trnfM–GCC*, *psbJ–psbL*, *trnW–CCA–petG*, *psbN–psbH*, and *ndhG–ndhE*, almost all other regions had variation. Previous studies have shown that *trnH*–*psbA*, *rps16*–*trnQ* (Dong et al., 2012), *atpH–atpI*, and *psaA–ycf3* (Cui et al., 2020) can be used as DNA barcodes in other plant taxa. Further studies are needed to confirm whether these CNS regions can be used to identify closely related species in *Astragalus*. These highly variable regions may also resolve the interspecific relationships of *Astragalus* in the legume phylogeny. *A. adsurgens*, *A. mongholicus* var. *dahuricus*, and *A. melilotoides* had lower levels of divergence concerning non-coding regions. However, there was less variation in the coding than in the non-coding regions. To further clarify the variation in the coding regions, Pi was also calculated (Figure 5). *atpB*, *psbD*, *rpoB*, *rpoC1*, *trnV*, *rrn16*, and *rrn23* all had high Pi values, exceeding 0.75. *atpB* and *psbD* encode proteins involved in photosynthesis, in which transcription is affected by light conditions; accordingly, high Pi values may reflect adaptation to different environmental light conditions (Christopher and Mullet, 1994). These highly variable regions may also resolve the interspecific relationships of *Astragalus* in the legume phylogeny.

### Selection on Functional Genes

**FIGURE 6:**
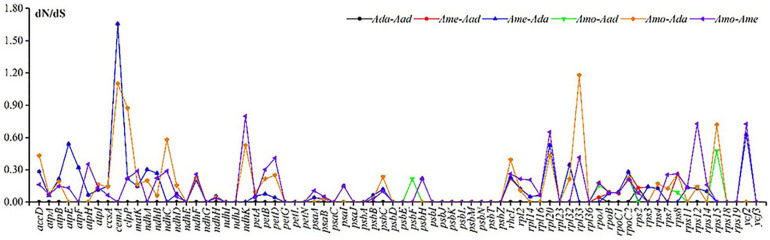
The non-synonymous/synonymous substitution rates (dN/dS) calculated using 74 shared genes in four *Astragalus* species.

The synonymous substitution rates (dS) of the four species in *Astragalus* ranged from 0.0000 to 0.0280 (*ycf2*), and the non-synonymous substitution rates (dN) ranged from 0.0000 to 0.0752 (*psbZ*). The ω value for 74 shared protein-coding genes within the species showed that *cemA* (encoding an envelope membrane protein) and *rpl33* (encoding the ribosomal protein L33) underwent positive selection (ω > 1), with the highest ω values (1.6545) being identified for *cemA* between *A. melilotoides*–*A. adsurgens* and *A. melilotoides*–*A. mongholicus* var. *dahuricus* (Figure 6 and Supplementary Table 4). The dN/dS ratio (ω) in the chloroplast genome provides important insights into adaptive molecular evolution (Dos Reis, 2015). The substitution rates in the cp genome are affected by both lineage-specific and locus-specific events; additionally, rate heterogeneity is mainly related to non-synonymous substitutions (Muse and Gaut, 1994). Synonymous variation is low in the cp genome; however, rates of non-synonymous changes are lower than those of synonymous changes (Volff et al., 2008), and most protein-coding genes related to photosynthesis undergo purifying selection (Jin et al., 2016). Positive selection based on high dN/dS substitution ratio is rare (Endo et al., 1996). Our results are consistent with these previous findings. Genes undergoing positive selection are mainly self-replication genes and those with unknown functions (Hong et al., 2020). In addition, rearrangements in the chloroplast genome may be subjected to positive selection (Sanderson and Doyle, 1993).

### Comparative Genome Analysis and Sequence Variation

**FIGURE 7:**
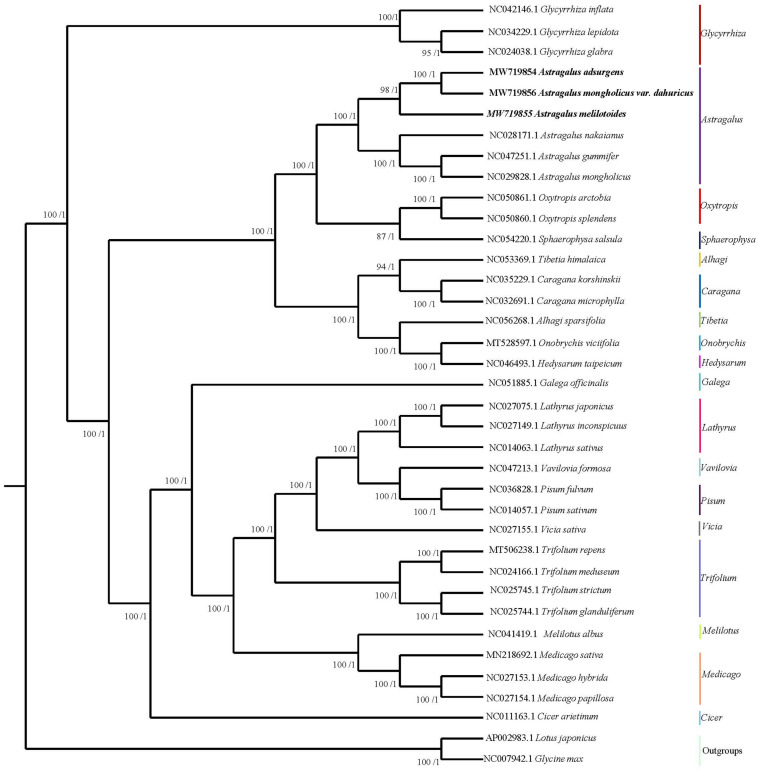
Phylogenetic tree of 35 species in Papilionoideae generated using maximum likelihood and Bayesian inferences (*Lotus japonicus* and *Glycine max* were used as outgroups) based on their complete cp genomes. Numbers associated with branches are ML values/BI values (posterior probability).

The topological structure of the phylogenetic tree of 35 species belonging to 18 genera in Papilionoideae as well as *L. japonicus* and *G. max*, which were used as outgroups, was consistent with the classification of Papilionoideae with strong bootstrap support (Figure 7). Six species of *Astragalus* formed a well-supported clade that included two major groups. *A. adsurgens* and *A. mongholicus* var. *dahuricus* showed the closest relationship among all *Astragalus* spp. Additionally, the genus *Astragalus* was monophyletic (Sanderson and Doyle, 1993; Wojciechowski et al., 1993) and was closely related to the clade that comprises the *Oxytropis* genus (Zimmers et al., 2017) and *Sphaerophysa salsula* within the Galegeae tribe. Previous studies have shown that there are 10 clades within *Astragalus*, including a new one, Pseudosesbanella, recovered in a recent phylogenetic analysis of coding sequences (Azani et al., 2019; Su et al., 2021). Our results confirm that *A. mongholicus* and *A. nakaianus* are in the Cenentrum section of Phaca, and *A. melilotoides* with *A. mongholicus* var. *dahuricus* belong to different sections (Su et al., 2021). The results of our phylogenetic analysis add to knowledge of previous studies and indicate that the cp genome can be used to construct relationships among species in this genus.

## Conclusion

In the present study, we sequenced and annotated the cp genomes of *A. adsurgens*, *A. mongholicus* var. *dahuricus*, and *A. melilotoides* in Papilionoideae (Leguminosae). All these species belong to the IRLC, and their genomes include repeat sequence and abundant SSRs. Using *A. membranaceus* var. *membranaceus* as reference, the divergence was evident in most coding regions of cp genomes of *Astragalus*, and seven genes can be used as candidate DNA barcodes. Most protein-coding genes undergo purifying selection, and only *cemA* and *rpl33* are under positive selection. *Astragalus* is a monophyletic group and is closely related to *Oxytropis*. Our analysis provides useful information for the identification and phylogenetic analyses of the IR lacking species.

## Data Availability Statement

The datasets presented in this study can be found in online repositories. The names of the repository/repositories and accession number(s) can be found below: https://www.ncbi.nlm.nih.gov/, SRR13870432, SRR13870430, and SRR13870431.

## Author Contributions

CT collected the plant materials, did the analysis, and wrote the first manuscript. ZW designed the experiment and performed data analysis. XL, ZL, XH, and FL contributed to the result interpretation and manuscript revision. All authors read and agreed to the published version of the manuscript.

## Conflict of Interest

The authors declare that the research was conducted in the absence of any commercial or financial relationships that could be construed as a potential conflict of interest.

## Publisher’s Note

All claims expressed in this article are solely those of the authors and do not necessarily represent those of their affiliated organizations, or those of the publisher, the editors and the reviewers. Any product that may be evaluated in this article, or claim that may be made by its manufacturer, is not guaranteed or endorsed by the publisher.

## Footnotes

1
https://chlorobox.mpimp-golm.mpg.de/OGDraw.html

2
https://webblast.ipk-gatersleben.de/misa/
